# Effectiveness of corridors varies among phytosociological plant groups and dispersal syndromes

**DOI:** 10.1371/journal.pone.0199980

**Published:** 2018-07-11

**Authors:** Jan Thiele, Jens Schirmel, Sascha Buchholz

**Affiliations:** 1 Institute of Landscape Ecology, University of Münster, Münster, Germany; 2 Thünen Institute of Biodiversity, Braunschweig, Germany; 3 Institute for Environmental Science, University of Koblenz-Landau, Landau, Germany; 4 Department of Ecology, Technische Universität Berlin, Berlin, Germany; 5 Berlin-Brandenburg Institute of Advanced Biodiversity Research (BBIB), Berlin, Germany; Charles University, CZECH REPUBLIC

## Abstract

In agricultural landscapes, semi-natural habitats are scarce and remaining habitat patches are largely isolated. However, linear landscape elements might facilitate dispersal of plant species through the agricultural landscape matrix. We investigated the following research questions: 1. are open linear landscape elements (LLE) effective corridors for dispersal of vascular plant species? 2. Which plant species, with respect to phytosociological group and dispersal-distance class, do use LLE as corridors? 3. To which extent is floristic similarity of communities influenced by dispersal through corridors? Field work was carried out in agricultural landscapes of Northwest Germany. We sampled 50 vegetation relevés on open linear landscape elements i.e. field margins (incl. road verges) and ditches, in eight 1-km^2^ study areas. Then, we calculated Jaccard similarities of all plot pairs within study areas using either all species or only species of certain phytosociological groups or dispersal-distance classes. We assessed the isolation of the plots from each other using both Euclidean distance and resistance distance along LLE. Resistance distance reflected the degree of connectivity of the LLE network between the plots. A stronger effect on Jaccard similarity of resistance distance compared to Euclidean distance would indicate corridor dispersal of plants through LLE. Relationships between Jaccard similarity and the two isolation measures were analysed with Generalised Linear Mixed Models. Resistance distance of LLE had a stronger negative effect on Jaccard similarity than Euclidean distance in field margins, but not in ditches. This was found for species of ‘meadows and pastures’ and short to medium dispersal distance. In plot pairs that were highly connected by LLE, the models suggested that roughly 20% of all species occurred in both plots due to dispersal through LLE. Other species groups did not respond more strongly to resistance distance than to Euclidean distance. We conclude that linear landscape elements in agricultural landscapes are effective corridors for dispersal of plant species that are confined to semi-natural habitats, such as traditional grasslands, and lack mechanisms of long-distance dispersal.

## Introduction

Intensification of land use has caused tremendous loss of semi-natural habitats, such as unimproved grasslands or wetlands. This has triggered a severe decrease of biodiversity in agricultural landscapes [1–3]. Next to the loss of area, fragments of these habitats have also become increasingly isolated in intensified agricultural landscapes which impedes dispersal and colonisation processes of plants and animals and has led to population declines and local extinctions of species [4,5].

In fragmented habitats, long-term conservation of biodiversity requires maintenance of connectivity between habitat patches [6,7]. Connectivity is necessary to enable gene flow and recolonisation of habitats after local extinctions and to facilitate range shifts in response to altered climate conditions [8,9]. Recovery or enhancement of connectivity can be achieved by providing corridors that facilitate dispersal (dispersal corridors) or serve as additional habitat (habitat corridors) and, thus, enable migrations of species between patches over several generations [10]. Linear landscape elements (LLE), such as field margins, ditches and hedges, may function as refuge habitats for species of traditional grasslands, wetlands and other semi-natural habitats [11–18]. Hence, they might also serve as habitat corridors for such species and, thus, provide connectivity between core habitat patches.

Several studies have given evidence for dispersal through corridors [10,19,20,21]. However, the effectiveness of corridors varies among species and taxonomic groups [10,22]. Previous studies mostly focussed on single or few species [10]. Thus, it remains largely unknown for which species a given type of corridor can provide functional connectivity, in the sense of effective dispersal of propagules or pollen [6,23,24]. Variation in corridor effects depends on species’ habitat preferences and dispersal capacities, but no consistent patterns of relationships between corridor efficiency and species' characteristics could be identified until now [10]. Thus, it is necessary to study a broad spectrum of species of a given community and to build more specific models for groups of species with similar dispersal abilities and habitat requirements [14,22,24,25].

Regarding plants, it is almost impossible to directly observe dispersal of propagules or multi-generational migration (hereafter subsumed under ‘dispersal’). Hence, it is necessary to use proxies for dispersal between sites in order to model functional connectivity. The most accurate proxy is genetic similarity of plant populations, but its application to whole communities is limited by the high analysis costs [26]. Thus, alternative proxies are required in order to develop a comprehensive theory of corridor ecology. In the present study, we used floristic similarity (Jaccard index) of vegetation relevés that were located on open LLE–field margins and ditches–in agricultural landscapes of Northwest Germany as a proxy for dispersal events that occurred between the compared plots.

Corridor networks, such as LLE in agricultural landscapes, may provide multiple paths for dispersal between two plots or habitat patches. Hence, classical least-cost distance [27] may not capture the total connectivity because it only considers single connections [28]. Therefore, we used resistance distance based on circuit theory [29] in order to model the connectivity of the LLE network. Resistance distance takes multiple connections into account so that overall resistance between two plots decreases with the number of potential dispersal pathways between them [30]. Resistance is the inverse of connectivity. Thus, a negative effect of resistance on floristic similarity would indicate a positive effect of connectivity.

In the present study, we used a new approach that combines assessment of corridor connectivity based on circuit theory (resistance distance) with using floristic similarity of communities as a proxy for dispersal. We aimed at answering the following questions:

- Are open LLE effective corridors for dispersal of vascular plants?
- Which plant species, with respect to phytosociological group and dispersal-distance class, can use open LLE as habitat corridors?
- To which extent is floristic similarity of communities influenced by dispersal through corridors?

## Methods

### Study areas

**Fig 1:**
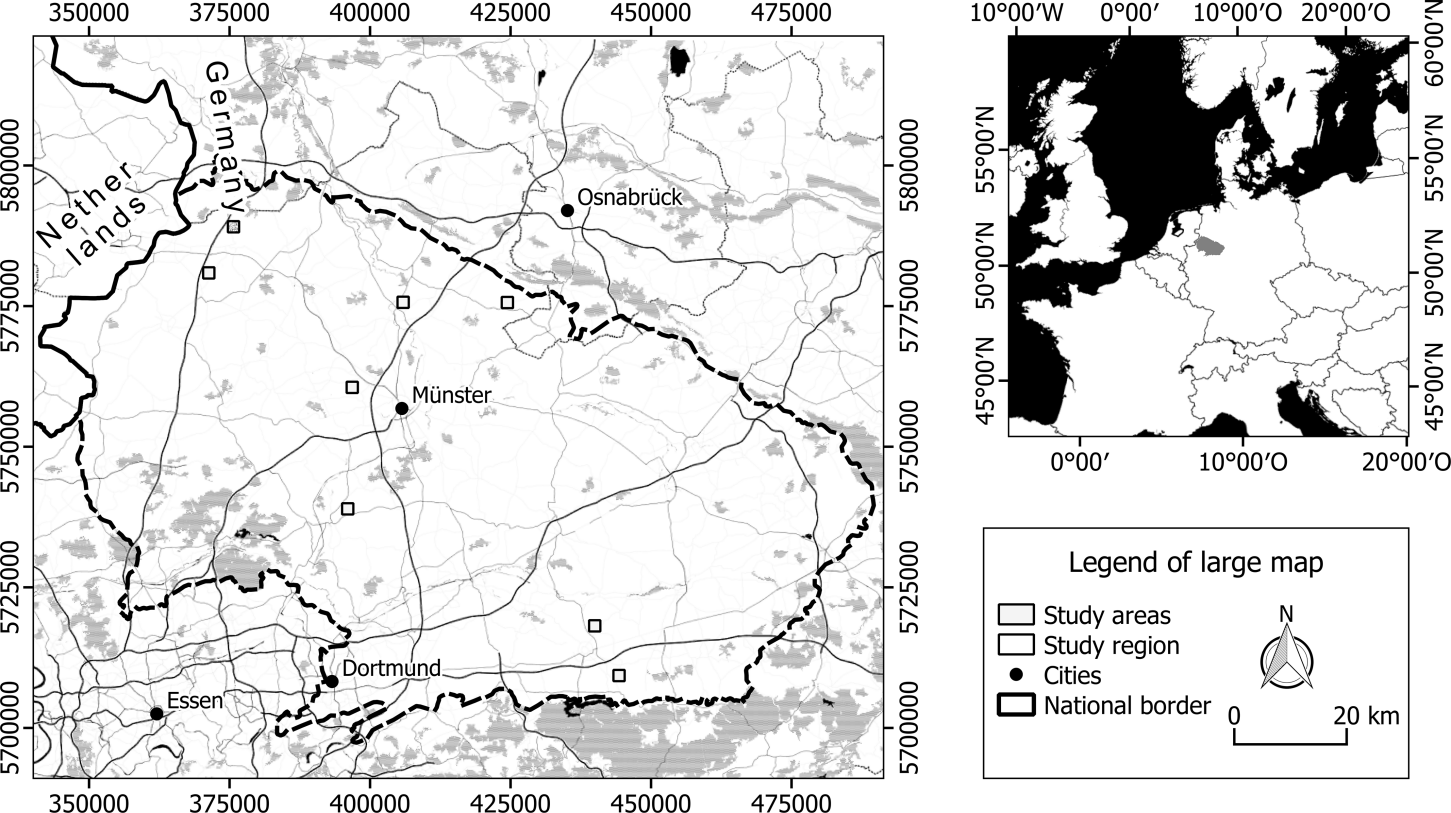
Locations of study areas. Small map: grey shading indicates the study region ‘Münsterland’ in Northwest Germany. Large map: locations of areas (squares) within the study region (Coordinate system: UTM 32N); grey shading represents larger forest areas. Base maps: map tiles by Stamen Design, under CC BY 3.0; data by OpenStreetMap, under CC BY SA.

The eight study areas (quadrats of 1 km^2^) were located in Northwest Germany (Fig 1; S1 Table). The climate of the region is temperate oceanic. The mean annual temperature is 10°C and annual precipitation is 800 mm (period 1981–2000; Klimaatlas Nordrhein-Westfalen, http://www.klimaatlas.nrw.de). Most of the study areas were located in lowlands at 40–80 m a.s.l., while the most southerly one was in slightly hilly terrain at 200–220 m a.s.l. Soil textures varied between sand in the western and northern parts, loam in the central part and loess in the southern part of the region, but all soils were lime-free.

The intensity of agricultural land use is very high in the study region and large proportions of permanent grasslands have been ploughed up during recent decades. The mowing frequency of remaining grasslands is 4–6 times per year. Thus, only extremely mowing-tolerant plant species (e.g. *Lolium multiflorum*, *L*. *perenne*, *Trifolium repens*) persist there, while most typical grassland species have disappeared. Also arable fields are subject to very intensive land use, including massive yearly application of herbicides and frequent tilling. For those reasons, most plant species do not find suitable habitat in the agricultural landscape matrix.

While all study areas face the same intensity of land use, they vary in the density of LLE, such as ditches, field margins, hedges and tree rows. Some of the study areas had a rather dense network of LLE, similar to bocage landscapes (mixed arable fields/ pastures and woodland), while others were more open with large fields and few LLE. The areal proportions of LLE varied between 2.5 and 10% of the total area so that there was a considerable density gradient.

The study areas were selected by random points in ArcGIS 10.1 (Environmental Systems Research Institute) using Geospatial Modelling Environment (GME) 0.7.2.1 (H.L. Beyer, http://www.spatialecology.com/gme). We intended to sample typical agricultural landscapes of the study region and, therefore, excluded areas dominated by other land-use or land-cover types, such as settlements, forests, lakes and rivers, or characterised by markedly different geology, e.g. lime stone, with the help of soil maps and Corine Land Cover (http://www.eea.europa.eu/publications/COR0-landcover).

### Field data

We surveyed 5–8 plots per study area (N_total_ = 50) located on open (treeless) LLE that we categorised into two classes: field margins (incl. road verges) and ditches. The selection of plot locations was again done with GME in ArcGIS 10.1 using random points that were located on LLE of at least 2 m width. The minimum distance between plots was established at 100 m to avoid spatial autocorrelation. The number of plots varied among study areas because some locations were highly disturbed or located on private land with forbidden access.

We sampled vegetation relevés [31] according to the Braun-Blanquet method recording all vascular plant species. As most LLE are only few meters wide, we established plots of 1 × 25 m^2^. The plots were placed on the centres of field margins. With respect to ditches, the placement of the plots depended on the width of the element. If the ditch was around 1 m wide (and usually dry during summer), the plot was placed on the centre. If the width was between 1 and 2 m, the plot was aligned with the shoulder of the embankment on one randomly selected side and, thus, covered the slope and part of the bottom of the ditch. Wider ditches (up to 5 m width) that contained water year round occurred occasionally. In these cases, the plots extended from the edge of the water surface upwards. The plots were visited in spring and in summer of 2012. Regarding plant communities, we found mesic grasslands (phytosociological order Arrhenatheretalia [32]) that additionally contained typical plant species of nitrophilous tall-herb communities (order Glechometalia) on the field margins. On ditches, the plant communities were transitional between mesic and wet grasslands (Molinietalia) and also comprised some species typical of fens and marshes. The species richness of plots was 22.6 (SD ± 7.6) on field margins and 24.6 (SD ± 10.6) in ditches.

### Jaccard similarity as proxy of dispersal

For all pairs of plots within the study areas, we calculated the unweighted Jaccard similarity index (*J*) according to the formula:

$$J=\frac{c}{S}$$ (1)

where *c* is the number of common species and *S* the total number of species of the two vegetation plots. We calculated the Jaccard indices using both all species and only species that belonged to certain groups according to their habitat preferences and dispersal syndromes.

The rationale behind using Jaccard similarity as a proxy of dispersal is that the open LLE in the studied landscape represented relatively new plant communities that were formed in the course of reallocation and consolidation of land in the 1960–70s or heavily modified through land-use intensification in that period. Thus, the floristic similarity of the communities was supposedly comparatively low due to quasi-randomness of colonisation and low initial species richness. Since then, we assumed, floristic similarity has increased until today due to dispersal between relevé plots. Then, if LLE were effective dispersal corridors, higher connectivity of such elements would result in higher floristic similarity. In general, floristic similarity of plots decreases with spatial distance (distance decay) either due to environmental differences or due to spatial and temporal constraints to dispersal [33]. Bearing this in mind, evidence for corridor function would be provided if the effect of connectivity of LLE on floristic similarity was larger than the effect of Euclidean distance.

### Phytosociological groups and dispersal-distance classes

In order to classify species according to their habitat preference, we categorised all species that are characteristic of a plant community on any level of the syntaxonomical system (alliance, order, class) according to Ellenberg et al. [34] into phytosociological groups (S2 Table). Some species that were not classified as characteristic species of any plant community in [34], but that we considered to be typical companion species were also assigned to the respective phytosociological groups (cf. S3 Table). The phytosociological groups used in this study were: species of *aquatic communities*, *fens and bogs*; *arable-weed*, *trackside and wasteland communities*; *meadows and pastures*; *nitrophilous tall-herb communities*; *nutrient-poor grasslands and heath*; and *wet grasslands and dwarf rush communities*.

With respect to dispersal syndrome, we grouped the species into three classes of terrestrial (i.e. non-aquatic) dispersal distance: short-, medium- and long-distance dispersal. The category “short-distance dispersal” was comprised of species with barochorous dispersal, seeds shed from capsules, or only aquatic dispersal mode, assuming that their propagules would usually not be dispersed further than several meters. “Medium-distance dispersal” comprised species with modes of wind or animal dispersal that would usually transport the seeds some decametres, e.g. myrmechory, while “long-distance dispersal” encompassed modes of wind and animal dispersal that facilitate dispersal over hundreds of metres or kilometres (S2 Table). Finally, we also grouped species with aquatic dispersal modes in another class that overlapped with dispersal-distance classes as some species have both aquatic and terrestrial dispersal modes. The classifications of dispersal groups were done according to [35] and, if species were not classified there, according to other sources as detailed in S2 Table.

### Resistance and Euclidean distance between plot pairs

As a baseline measure of isolation, we calculated the Euclidean distance between all possible pairs of vegetation plots within study areas in ArcGIS 10.1. Further, we assessed the connectivity of LLE between the plots using resistance distance based on circuit theory [29,30]. Resistance distance is a measure of isolation and, thus, is the reciprocal of connectivity. In our study, resistance distance showed a moderate correlation with Euclidean distance of r = 0.48 (p < 0.001).

First, we mapped all LLE, including those containing woody elements (i.e. hedges, tree rows and shaded ditches) and forest edges (inner buffers of forests of 2 m width), based on aerial images with 20 cm resolution available from the state’s land surveying office in ArcGIS 10.1. The maps covered buffers of 500 m around the study areas to prevent bias in the estimates of resistance distance due to boundary effects [36]. We assigned an arbitrary resistance value of 0.01 to all LLE polygons (i.e. conductivity was set to 100). Small gaps (< 10 m) among LLE were closed by using buffers of 5 m around the mapped LLE polygons as we assumed that they could occasionally be bridged even by short-distance dispersed species. However, we also assumed that small gaps decreased the likelihood of dispersal. Therefore, we assigned doubled resistance values (0.02) to the buffer areas. This parameterisation was shown to perform better than variants with full connectivity of small gaps [28].

Then, the LLE maps were converted to rasters with 1-m resolution where all cells located on LLE or buffers contained the respective resistance values, whereas cells in the landscape matrix received infinite resistance (zero conductivity). This parameterisation of cell-level resistances reflected the hypothesis that LLE are habitat corridors for multi-generational migration of plants, whereas the landscape matrix is inhospitable and, thus, prevents migration. Based on the resistance rasters, we calculated the effective resistance distance of the LLE network between each pair of relevé plots using the plugin ‘Circuitscape for Processing 0.1.1’ (https://github.com/alexbruy/processing_circuitscape) in QGIS 2.6 [37].

### Statistical analyses

We modelled the effects of Euclidean distance and resistance distance on Jaccard similarity of relevé plots with Generalised Linear Mixed Models (GLMM) using the function *glmer* of the *lme4* package [38] in R version 3.3.1 [39]. The sample used for analysis included 50 plots and 132 observations (i.e. ‘pairs’). The dependent variable, Jaccard similarity, was coded as proportion data except for the species groups *arable-weed*, *trackside and wasteland communities* and *aquatic dispersal* that contained many zero values (S4 Table) and, thus, were coded as binary data (‘0’ if Jaccard similarity was 0, or ‘1’ if Jaccard similarity was > 0). All models were set up with binomial distribution and logit link. The predictor variables (fixed effects) comprised, firstly, either Euclidean or resistance distance, secondly, the co-factor ‘LLE types’ which coded the types of LLE that were compared (three levels: margin-margin, margin-ditch, ditch-ditch) and, thirdly, the interaction of the respective isolation measure with LLE types. Isolation measures were z-transformed (centred and scaled). The random-effect structure of the models included study area (eight levels) because of the nested sampling design. Further, the ‘pairs’ of relevés were certainly not statistically independent as each plot occurred in several pairs. Therefore, we included random effects of ‘plot A’ and ‘plot B’, i.e. the first and the second plot of the pair. If the models showed overdispersion, we also included an ‘individual-level’ random effect [40], cf. http://bbolker.github.io/mixedmodels-misc/glmmFAQ.html. This was the case with all but the binary models (S4 Table). Some phytosociological groups could not be analysed because they showed too few non-zero values of Jaccard similarity (*nutrient-poor grasslands and heath*), or the models would not converge and yield unrealistic coefficients and standard errors (*aquatic communities*, *fens and bogs*, *wet grasslands and dwarf rush communities*) (S4 Table).

We tested for linearity of the relationship between resistance distance and Jaccard similarity using the *cumres* function (with default settings) of the *gof* package [41] in R. For this purpose, we calculated Generalised Linear Models (GLM) that included a fixed effect of study area in addition to the other fixed effects (but excluded the observation-level effects), as *cumres* does not accept GLMM. We did not find severe deviation from linearity in any of the models.

The main effects of the GLMM were tested for significance using parametric bootstrap with 999 replications. We tested the simple regression slopes within each combination of LLE types for significant difference from zero using two-tailed t-tests. With the slopes of the baseline category (“margin-margin”), we used the estimate and standard error of the main effect of the isolation measure in order to calculate the test statistics and p-values. The slopes of the other two LLE types were calculated as the estimate of the main effect plus the estimate of the respective interaction effect, and the corresponding standard errors were calculated using the formula:

$${se}_{slope}=\sqrt{{se}_{main}^{2}+2\times cov(main,int.)+{se}_{int.}^{2}}$$ (2)

where *se*^2^ is the variance (squared standard error) of the respective regression coefficient (main effect or interaction effect) and *cov* is the covariance between main and interaction effect. As degrees of freedom are a controversial issue with GLMM, we calculated two variants supposed to represent the maximum and minimum possible degrees of freedom: *df*_max_ = *N*-*p*-*g*-1 and *df*_min_ = *n*-*p*-*g*-1, where *N* is the total sample size (i.e. number of pairs of vegetation plots, 132), *n* is the number of plots used in this study (49), *p* is the number of fixed effects parameters in the model (6), and *g* is the number of grouping levels in the random factors ‘study area’ (8). In all cases, the two variants of degrees of freedom gave unequivocal results. The conservative p-values based on the minimum degrees of freedom are reported in the results.

Differences between slopes of Euclidean and resistance distance were tested with Z-tests according to the formula:

$$Z=\frac{b_{1}-b_{2}}{\sqrt{{{se}_{b1}}^{2}+{{se}_{b2}}^{2}}}$$ (3)

where *b*_*1*_ is the slope of resistance distance, *b*_*2*_ is the slope of Euclidean distance, and *se* denotes the standard error of the respective coefficient.

Finally, we compared predicted effects of Euclidean and resistance distance. For this purpose, we calculated the predicted increase of Jaccard similarity when Euclidean or resistance distance decreased from their maximum to their minimum. Then, we calculated the difference in the increase between the two isolation measures. The increase in the number of common species of the vegetation plots (*Δc*), given a certain increase in Jaccard similarity (*ΔJ*), depends on the cumulative species number (*S*) of the two vegetation plots that are compared as shown in the following:

$$\Delta J=J_{2}-J_{1}=\frac{c_{2}}{S}-\frac{c_{1}}{S}=\frac{\Delta c}{S}\underset{}{\Leftrightarrow }\Delta c=\Delta J\times S$$ (4)

Hence, we multiplied the predicted increases in Jaccard similarity with hypothetical, but realistic, numbers of total species in order to get an idea of the number of common species that were attributable to decreasing isolation.

## Results

Significant effects of isolation measures–either Euclidean distance or resistance distance or both–on Jaccard similarity were found for all species groups, except for species with aquatic dispersal where resistance distance, however, was close to significance level (p = 0.063; Table 1; detailed model output is given in S5 Table). Isolation effects were limited to comparisons of field margins with field margins, whereas there were no significant isolation effects within the other comparisons (margin with ditch and ditch with ditch; Table 1).

**Table 1:** Modelling results of Generalised Linear Mixed Models (GLMM) of Jaccard similarity vs. Euclidean distance and resistance distance for different phytosociological species groups and dispersal-distance classes.

	Euclidean distance			Resistance distance			Difference in effect estimates		
Species group/ level	b	se_b_	p	b	se_b_	p	delta	se_delta_	p_delta_
**All species**			**0.016**			**0.001**			
margin-margin	-0.439	0.190	**0.027**	-1.149	0.229	**< 0.001**	-0.709	0.298	0.017
margin-ditch	-0.110	0.217	0.614	-0.206	0.248	0.410	-0.096	0.329	0.770
ditch-ditch	0.021	0.258	0.935	-0.051	0.274	0.852	-0.073	0.376	0.847
**Meadow and pasture species**			**0.048**			**0.002**			
margin-margin	-0.632	0.364	*0*.*091*	-1.701	0.447	**< 0.001**	-1.068	0.577	*0*.*064*
margin-ditch	-0.166	0.412	0.690	-0.391	0.481	0.422	-0.225	0.634	0.722
ditch-ditch	-0.034	0.490	0.945	0.022	0.532	0.967	0.056	0.723	0.938
**Tall-herb communities**			**0.046**			*0*.*062*			
margin-margin	-0.501	0.328	0.135	-0.655	0.456	0.160	-0.154	0.562	0.784
margin-ditch	-0.174	0.375	0.646	-0.192	0.493	0.699	-0.018	0.620	0.977
ditch-ditch	0.007	0.446	0.988	-0.097	0.549	0.861	-0.103	0.707	0.884
**Weeds and trackside species**			0.231			**0.021**			
margin-margin	0.385	0.789	0.629	-1.440	1.300	0.275	-1.825	1.521	0.230
margin-ditch	-0.468	0.894	0.604	-0.480	1.381	0.730	-0.012	1.645	0.994
ditch-ditch	-0.292	1.076	0.787	-1.607	1.815	0.382	-1.315	2.110	0.533
**Short-distance dispersal**			**0.010**			**0.001**			
margin-margin	-0.777	0.289	**0.011**	-1.585	0.361	**< 0.001**	-0.808	0.462	*0*.*080*
margin-ditch	-0.215	0.326	0.513	-0.376	0.387	0.339	-0.160	0.506	0.752
ditch-ditch	0.093	0.388	0.811	-0.129	0.162	0.765	-0.222	0.421	0.597
**Medium-distance dispersal**			0.176			0.165			
margin-margin	-0.850	1.623	0.604	-5.614	2.034	**0.009**	-4.764	2.603	*0*.*067*
margin-ditch	-0.418	1.857	0.823	0.149	2.183	0.946	0.567	2.866	0.843
ditch-ditch	-1.648	2.216	0.462	-1.592	2.435	0.517	0.056	3.292	0.987
**Long-distance dispersal**			0.365			*0*.*093*			
margin-margin	-0.398	0.240	0.106	-0.684	0.330	**0.045**	-0.286	0.408	0.483
margin-ditch	-0.011	0.274	0.969	-0.089	0.356	0.804	-0.078	0.449	0.862
ditch-ditch	0.069	0.327	0.833	0.102	0.394	0.797	0.033	0.513	0.949
**Aquatic dispersal**			0.201			*0*.*063*			
margin-margin	-0.312	0.674	0.646	-1.674	0.960	*0*.*090*	-1.362	1.173	0.246
margin-ditch	-0.098	0.770	0.900	-0.224	1.017	0.827	-0.126	1.275	0.921
ditch-ditch	-0.708	0.923	0.448	-0.496	1.109	0.658	0.213	1.443	0.883

Significance tests of the main effects were conducted with parametric bootstraps of the GLMM. Simple slopes of Euclidean and resistance distance within particular combinations of types of linear landscape elements (“margin-margin” etc.) were tested for significance using t-tests. Differences in effect estimates between the Euclidean and resistance distance were tested with Z-tests. Abbreviations: b = regression coefficient (estimate), se = standard error, p = significance level, delta = difference between regression coefficients of resistance and Euclidean distance.

**Fig 2:**
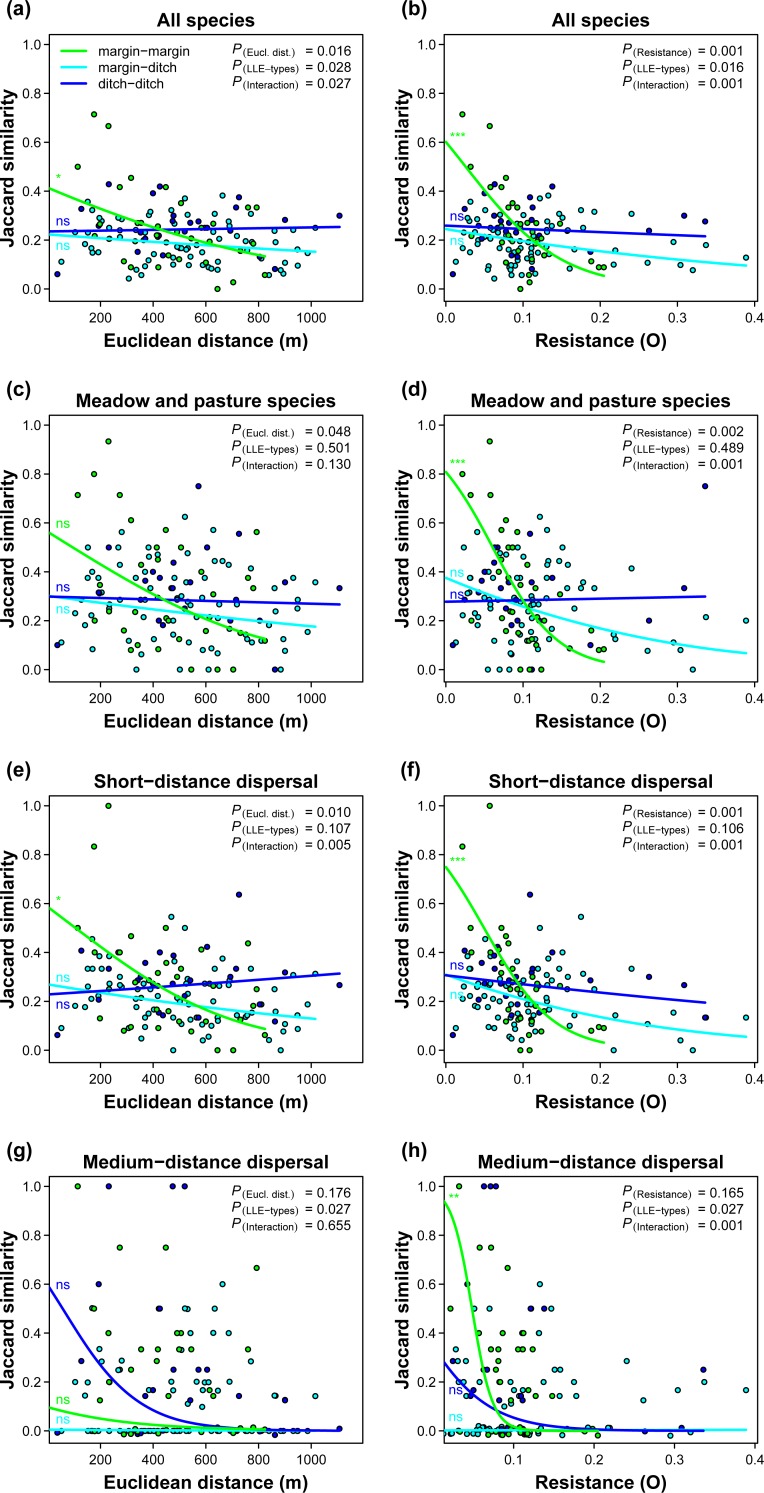
Jaccard similarity vs. the isolation metrics Euclidean distance and resistance distance of linear landscape elements. Jaccard similarity was calculated for different species groups: (a, b) all vascular plant species, (c, d) species typical of *meadows and pastures*, (e, f) species with short-distance dispersal, and (g, h) species with medium-distance dispersal (cf. S2 Table). There were two types of LLE (field margins and ditches) and, consequently, three combinations of LLE types in the calculations of Jaccard similarity: margin compared with margin, margin compared with ditch, and ditch compared with ditch. Prediction curves are from binomial Generalised Linear Mixed Models (GLMM). Inset p-values (upper right corner) are from parametric bootstrap tests of the GLMM. Significance levels of simple regression slopes within LLE-type combinations: ns, not significant; *, p < 0.05; **, p < 0.01; ***, p < 0.001.

Resistance distance had a stronger negative effect on Jaccard similarity compared to Euclidean distance with respect to *all species* (p = 0.017), the phytosociological group of *meadows and pastures* (p = 0.064), and the dispersal-distance classes of *short- and medium-distance dispersal* (p = 0.080 and 0.067, respectively), but only when two field margins were compared (Table 1; Fig 2). Species of *arable-weed*, *trackside and wasteland communities* and species with *aquatic dispersal* mode showed a tendency towards stronger effects of resistance distance compared to Euclidean distance, but the differences in effect size were not significant. Species of *nitrophilous tall-herb communities* showed weak response to both Euclidean distance and resistance distance. *Long-distance dispersed* species showed a marginally significant effect of resistance distance, but not Euclidean distance. However, the difference in effect between the two isolation measures was far from being significant.

As the effects of isolation measures were negative, predicted Jaccard similarity increased when resistance and Euclidean distance decreased from their maximum to minimum values. Regarding the species groups where resistance distance had a more negative effect size than Euclidean distance, the models predicted, thus, an additional increase of Jaccard similarity over and above the effect of decreasing spatial isolation. With respect to *all species*, the additional increase in Jaccard similarity was 0.23 which would translate into additional 8 common species given that the two compared relevés had a cumulative species number of 36 which was the mean. For the other pertinent groups, the number of additional common species was between 3–6 (Table 2).

**Table 2:** Predicted Jaccard similarities of relevés located on field margins at minimum and maximum values of Euclidean distance and resistance distance.

	Euclidean			Resistance			Δ Incr.	Δ Spec.	
	Min	Max	Incr.	Min	Max	Incr.			Mean S
All species	0.36	0.13	0.23	0.52	0.05	0.46	0.23	8	36
Meadow and pasture species	0.49	0.13	0.36	0.72	0.03	0.69	0.32	5	17
Short-distance dispersal	0.49	0.09	0.41	0.65	0.03	0.62	0.21	3	15
Medium-distance dispersal	0.07	0.01	0.06	0.89	0.00	0.89	0.83	4	5

The predicted increase (“Incr.”) of Jaccard similarity from maximum to minimum distance/ resistance, and the difference in predicted increase between the two isolation measures (“Δ Incr.”) are given. The differences in increase of Jaccard similarities were translated into numbers of additional common species (“Δ Spec.”) based on the mean cumulative species richness (“Mean S”) of relevé pairs within the respective species group.

## Discussion

Within field margins, resistance distance had a stronger effect on Jaccard similarity than Euclidean distance with respect to *all species*, the phytosociological group of *meadow and pasture species*, and the dispersal classes of *short-distance dispersal* and *medium-distance dispersal*. Even though statistical tests of difference in effect sizes between resistance and Euclidean distance were slightly above the standard significance level of 0.05 with the latter three groups of species, we would consider the effect of resistance distance to be stronger because of markedly larger (i.e. more negative) effect estimates (Table 1) and substantially higher predicted numbers of common species (Table 2).

**Fig 3:**
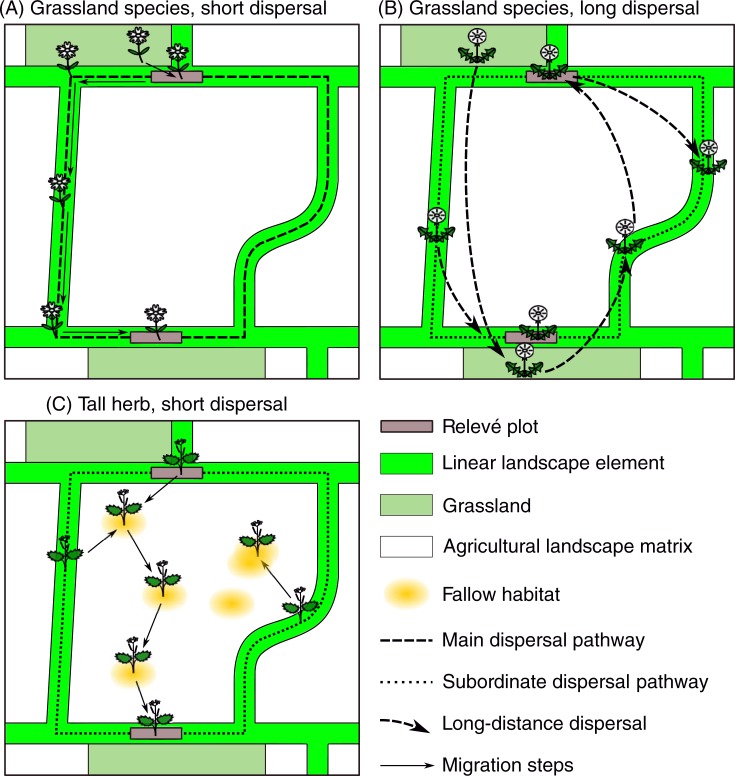
Dispersal pathways deduced from statistical results for different types of plants. (A) Species confined to semi-natural grasslands and dispersed only short distances rely on multi-generational migration through Linear Landscape Elements (LLE). (B) Grassland species with mechanisms of long-distance dispersal (wind, animals) mainly disperse through the agricultural landscape matrix, while migration through LLE is less important. (C) Nitrophilous tall herbs find habitat on small fallow spots within the landscape matrix, and, thus, disperse through the matrix by stepwise migration from one fallow habitat to the next, if they lack mechanisms of long-distance dispersal.

From the stronger effects of resistance distance on floristic similarity, we conclude that LLE are effective corridors for dispersal of plant species that lack capabilities for long-distance dispersal and are confined to semi-natural open habitats in agricultural landscapes (Fig 3A). Thus, LLE can increase the functional connectivity between core habitat areas, while the landscape matrix (mainly arable fields, silage grasslands and forests) impedes dispersal and decreases connectivity for such species. Regarding the strength of the corridor effect, we found that eight common species out of 36 species (average cumulative species number of plot pairs) were attributable to decreasing resistance distance of LLE, beyond the distance-decay effect of Euclidean distance, when comparing predictions at maximum and minimum distance (Table 2). Thus, in plot pairs that are highly connected by LLE, the model suggests that roughly 20% of species occur in both plots due to dispersal through corridors. Hence, we would conclude that corridor dispersal is quantitatively important regarding both colonisation of core habitats and composition of plant communities with respect to the aforementioned plant groups.

Within ditches, we did not find effects of Euclidean distance or resistance distance on floristic similarity which is in contrast to recent studies that, in the majority, provided evidence for facilitation of plant dispersal through ditches [42–44] for negative evidence see [45]. The reason for lack of effect in this study, may lie in the parameterisation of resistance distance that did not consider if the ditch network between two plots was connective throughout or if there were breaks, such as culverts or interruptions, in between. More generally, resistance distance may fail to accurately assess ditch connectivity as it does not consider flow direction and it may be difficult to parameterize the resistance of culverts [44]. Another reason for lack of significant effect may be the relatively low number of species that supposedly would respond to ditch connectivity because they are confined to wet habitats (mean of ditch relevés: 6.0 ± 5.7 out of 55 species) and/ or dispersed by water (3.1 ± 2.6 out of 26 species of which four species occur only in terrestrial habitats).

In the literature, the majority of studies support corridor function of LLE [10,21]. However, the effectiveness of corridors varies among taxonomic and ecological groups of species [22]. In this study, we did not find a corridor effect for species of *nitrophilous tall-herb communities*. As fifty percent of species in this group do not have mechanisms of long-distance dispersal, this suggests that the landscape matrix provides habitat for species of this group. For instance, species such as *Aegopodium podagraria*, *Glechoma hederacea* or *Lapsana communis* do frequently occur on fallow land and waste ground in the study region, next to fringes along hedges and woodlands, or field margins. Thus, the landscape matrix apparently offers as much connectivity for such species as LLE do (Fig 3C).

Species of *arable-weed*, *trackside and wasteland communities* showed a significant effect of resistance distance, but did not respond to Euclidean distance. Statistically, the stronger effect of resistance distance was not significant which is attributable to the comparatively large standard errors in the respective model. Nevertheless, the results suggest that species of this group disperse more along LLE than through the landscape matrix which is remarkable because agricultural land is potential habitat for many of these species. However, it seems that the habitat quality of agricultural land is rather poor even for ruderal species which may be due to the highly intensive land use that includes frequent and abundant application of herbicides [46].

Species of *nutrient-poor grasslands and heath* occurred rarely in our dataset. While this made it impossible to build stable models, it clearly showed that the habitat quality of LLE in our study areas did not match the preferences of this species group, mainly because the nutrient load was too high due to massive fertiliser application on adjacent fields. Hence, the LLE fail to provide corridor function, and enhancement of connectivity would require to lower the nutrient status regarding species of nutrient-poor habitats.

As expected, species with mechanisms of long-distance dispersal (anemochory, endozoochory, epizoochory) did not show substantially stronger response to corridor connectivity compared to Euclidean distance [47]. It seems that even those species of this group that are confined to semi-natural habitats (54% of the species with long-distance dispersal), are effectively dispersed through the landscape matrix by their dispersal vectors (Fig 3B). Notwithstanding, resistance of LLE had a marginally significant effect on long-distance dispersed species in field margins suggesting that their dispersal is facilitated by corridors to some degree. This might be attributable to animal dispersers that preferentially move along LLE [14].

Resistance distance also depends on the area of the landscape elements that are considered to provide connectivity (here: LLE), next to the distance and the number of connecting elements between the compared plots [48]. Hence, it is in principle possible that statistical effects of resistance distance are partly due to variation in habitat area that may affect population sizes and, thus, dispersal likelihood. However, we think that area effects are unlikely in this study because the LLE and, in particular, the field margins were narrow (< 5 m) with little variation in width. Further, in a parallel study, we found no significant effect of LLE area, but of resistance distance, on species richness of the plots [49]. Likewise, in a meta-analysis of other studies, corridor effects were not confounded by area effects [10]. Thus, we are confident that the results show true effects of corridor connectivity.

Our approach to use community similarity of ‘new’ vegetation stands (formed some decades ago and still being colonised by plant species) for testing and modelling of corridor effects has got strengths and weaknesses. On the one hand, it is a disadvantage that floristic similarity does not capture all dispersal events as it does not reflect repeated dispersal events by species that were already common to both relevés. Hence, community similarity underestimates dispersal. The relationship between the number of dispersal events and the resulting increase in community similarity can be modelled with a limited growth function Supplementary Figure S1 in [28] which, theoretically, would allow to infer dispersal events from Jaccard similarity. However, this would require to know the initial species richness and similarity of the relevés and such data are rarely available.

On the other hand, community data such as vegetation relevés are easily acquired and highly available from databases. Thus, they may facilitate to study corridor effects with higher spatial and temporal repetition. In particular, the use of floristic similarity allows to study whole communities simultaneously and to test hypotheses on the response of ecological or functional species groups to corridor connectivity. Even though it is not possible to assess the exact number of dispersal events per unit time with this approach, it is possible to model the relative strength of connectivity effects of different landscape elements.

In comparison, population genetic indices measure dispersal or gene flow much more accurately, but the high sampling effort and costs of analysis largely prevent their application to multiple species groups, let alone whole communities [26]. Therefore, the use of floristic similarity, based on simple community data, could be a useful ad-hoc criterion for assessing connectivity among core habitat areas provided by habitat corridors or other landscape elements. Hence, it could contribute to improving the information basis for habitat network planning in order to increase resilience of plant communities by maintaining suitable dispersal pathways.

## Conclusions

LLE in agricultural landscapes are effective corridors for dispersal of plant species that are confined to semi-natural habitats (meadow and pasture species, in this study) and that lack mechanisms of long-distance dispersal. Thus, LLE can increase connectivity among core habitat areas and, in this way, help to sustain species of these groups in intensively used agricultural landscapes. In contrast, plant species that find habitat on agricultural land or disturbed sites (arable weeds, nitrophilous tall herbs) or that have mechanisms of long-distance dispersal, i.e. wind or animal dispersal (epi- and endozoochory), can disperse through the landscape matrix and, thus, do not benefit substantially from LLE. Floristic similarity is a useful proxy of dispersal in comparatively ‘new’ habitats where the distributions of species at landscape scale has not yet reached an equilibrium. Then, floristic similarity may sufficiently reflect the number of dispersal events between sites in order to model the connectivity provided by landscape elements.

## Supporting information

S1 Table Study areas. UTM (zone 32N) coordinates of centroids of study areas.
(DOCX)

S2 Table Species groups. Definitions of phytosociological groups and dispersal-distance classes.
(DOCX)

S3 Table Species list. Species list with assignments to phytosociological groups and dispersal-distance classes.
(DOCX)

S4 Table Model setup. Setup of Generalised Linear Mixed Models.
(DOCX)

S5 Table Model summaries. Summary tables of Generalised Linear Mixed Models.
(DOCX)

S1 File Dataset. (CSV)

S2 File Vegetation relevés. (CSV)

S1 Text Metadata. Metadata of the dataset used in the article.
(TXT)

S2 Text Script. R script of statistical analyses.
(R) Click here for additional data file.
