# Supplementary material for: Effectiveness of corridors varies among phytosociological plant groups and dispersal syndromes
Source: PLoS One. 2018 Jul 11;13(7):e0199980. doi: 10.1371/journal.pone.0199980 (PMC6040708; doi:10.1371/journal.pone.0199980)
Supplement: S1 Table — UTM (zone 32N) coordinates of centroids of study areas. (DOCX) [file pone.0199980.s001.docx]

Supporting information to the paper

Thiele, J., Buchholz, S. & Schirmel, J. (2018) Effectiveness of corridors varies among phytosociological plant groups and dispersal syndromes. Plos One.

**S1 Table. Study areas.** UTM (zone 32N) coordinates of centroids of study areas.

| Name | UTM East | UTM North |
| --- | --- | --- |
| Ammerter Mark | 371299 | 5780897 |
| Herringser Höfe | 444309 | 5709305 |
| Holzhausen | 424438 | 5775629 |
| Osterbauerschaft | 375697 | 5789104 |
| Rugenbusch | 396029 | 5738944 |
| Schonebeck | 396829 | 5760545 |
| Thöningsen | 439981 | 5718142 |
| Wentrup | 405885 | 5775669 |
