# Supplementary material for: Effectiveness of corridors varies among phytosociological plant groups and dispersal syndromes
Source: PLoS One. 2018 Jul 11;13(7):e0199980. doi: 10.1371/journal.pone.0199980 (PMC6040708; doi:10.1371/journal.pone.0199980)
Supplement: S2 Table — Definitions of phytosociological groups and dispersal-distance classes. (DOCX) [file pone.0199980.s002.docx]

Supporting information to the paper

Thiele, J., Buchholz, S. & Schirmel, J. (2018) Effectiveness of corridors varies among phytosociological plant groups and dispersal syndromes. Plos One.

**S2 Table. Species groups.** Definitions of phytosociological groups and dispersal-distance classes.

**Table S1.1.** Names of phytosociological groups used in this study and corresponding syntaxonomical units according to Ellenberg et al. (1992). Additionally, the number of species of each group in this study’s dataset is given.

| **Phytosociological group** | **Syntaxonomical units** | **No. of species** |
| --- | --- | --- |
| Aquatic communities, fens and bogs | 1.1 Lemnetea, 1.2 Utricularietea, 1.3 Potamogetonetea, 1.4 Littorelletea | 28 |
| Arable-weed, trackside and wasteland communities | 3.1 Isoëto-Nanojuncetea, 3.2 Bidentetea, 3.3 Chenopodietea, 3.4 Secalietea, 3.7 Plantaginetea | 33 |
| Meadows and pastures | 5.42 Arrhenatheretalia | 40 |
| Nitrophilous tall-herb communities | 3.5 Artemisietea, 3.6 Agropyretea, 6.1 Trifolio-Geranietea, 6.2 Epilobietea | 43 |
| Nutrient-poor grasslands and heath | 5.1 Nardo-Callunetea, 5.2 Sedo-Scleranthetea, 5.3 Festuco-Brometea | 14 |
| Wet grasslands and dwarf rush communities | 5.41 Molinietalia, 3.8 Agrostietea | 27 |

**Table S1.2.** Definitions of dispersal-distance classes (only non-aquatic dispersal) and corresponding dispersal syndrome after Hodgson et al. (1995). Species not classified there were assigned to dispersal syndromes based on Düll & Kutzelnigg (2011) or www.floraweb.de. Few species for which no unequivocal classification could be found were assigned based on expert opinion (cf. Table S2).

| **Dispersal-distance class** | **Dispersal syndromes after Hodgson et al. 1995** |
| --- | --- |
| Short-distance dispersal up to several meters | unspecific/ barochory UNSP;  wind, shed from capsules WINDc;  aquatic AQUAT |
| Medium-distance dispersal decametres | winged seeds/ fruits WINDw;  myrmechory ANIMe;  zoochory, nuts etc. ANIMn |
| Long-distance dispersal hundreds of meters to kilometres | wind, minute seeds WINDm;  wind, with pappi WINDp;  epizoochory, awn or calyx teeth ANIMa;  epizoochory, burr ANIMb;  epizoochory, sticky seed ANIMm  endozoochory, ingested berry ANIMi |

**References**

Düll R, Kutzelnigg H 2011 Taschenlexikon der Pflanzen Deutschlands und angrenzender Länder. Wiebelsheim: Quelle & Meyer.

Hodgson JG, Grime JP, Hunt R, Thompson K 1995 The electronic comparative plant ecology. London: Chapman & Hall.
