# Supplementary material for: Effectiveness of corridors varies among phytosociological plant groups and dispersal syndromes
Source: PLoS One. 2018 Jul 11;13(7):e0199980. doi: 10.1371/journal.pone.0199980 (PMC6040708; doi:10.1371/journal.pone.0199980)
Supplement: S3 Table — Species list with assignments to phytosociological groups and dispersal-distance classes. (DOCX) [file pone.0199980.s003.docx]

Supporting information to the paper

Thiele, J., Buchholz, S. & Schirmel, J. (2018) Effectiveness of corridors varies among phytosociological plant groups and dispersal syndromes. Plos One.

**S3 Table. Species list.** Species list with assignments to phytosociological groups and dispersal-distance classes.

**Table S2.** Classifications of plant species in phytosociological groups and dispersal-distance classes. Abbreviations of phytosociological groups: *aquatic communities, fens and bogs*: aqfb; *arable-weed, trackside and wasteland communities*: awtw; *meadows and pastures*: m&p; *nitrophilous tall-herb communities*: nth; *nutrient-poor grasslands and heath*: pg&h; *swamp woodland*: swood; *wet grasslands and dwarf-rush communities*: wgdr; *woodland*: wood. Sources of dispersal syndromes: Bruun: Bruun (2009); Düll: Düll & Kutzelnigg (2011); Floraweb: www.floraweb.de; Hodgson: Hodgson et al. (1995); Mersereau: Mersereau & DiTommaso (2003); authors: the authors’ own assessment; Wikipedia: article on the given species on www.wikpedia.org.

| **Species** | **Phytosocio-logical group** | **Dispersal distance class (non-aquatic)** | **Aquatic dispersal** | **Source of dispersal syndrome** |
| --- | --- | --- | --- | --- |
| Acer pseudoplatanus | wood | medium | no | Hodgson |
| Achillea millefolium agg. | m&p | medium | no | Hodgson |
| Aegopodium podagraria | nth | short | no | Hodgson |
| Aesculus hippocastanum | not assigned | medium | no | Floraweb |
| Agrimonia eupatoria | nth | long | no | Hodgson |
| Agrostis capillaris | m&p | short | no | Hodgson |
| Agrostis gigantea | m&p | short | no | Hodgson |
| Agrostis stolonifera | wgdr | short | no | Hodgson |
| Alisma plantago-aquatica | aqfb | long | yes | Hodgson |
| Alliaria petiolata | nth | short | no | Hodgson |
| Alnus glutinosa | swood | medium | yes | Hodgson |
| Alopecurus myosuroides | awtw | long | no | Floraweb |
| Anemone nemorosa | wood | long | no | Hodgson |
| Angelica sylvestris | wgdr | medium | yes | Hodgson |
| Anthoxanthum odoratum | pg&h | long | no | Hodgson |
| Anthriscus sylvestris subsp. sylvestris | m&p | short | no | Hodgson |
| Apera spica-venti | awtw | long | no | Floraweb |
| Arctium lappa | nth | long | no | Hodgson |
| Arenaria serpyllifolia subsp. serpyllifolia | pg&h | short | no | Hodgson |
| Arrhenatherum elatius | m&p | long | no | Hodgson |
| Artemisia vulgaris | nth | short | no | Hodgson |
| Athyrium filix-femina | wood | long | no | Hodgson |
| Betula pendula | wood | medium | no | Hodgson |
| Brassica napus | not assigned | short | no | authors |
| Bromus hordeaceus subsp. hordeaceus | m&p | long | no | Hodgson |
| Bromus inermis | nth | long | no | Floraweb |
| Bromus racemosus | wgdr | long | no | Floraweb |
| Bromus sterilis | awtw | long | no | Hodgson |
| Callitriche palustris agg. | aqfb | long | yes | Floraweb |
| Caltha palustris | wgdr | short | yes | Hodgson |
| Calystegia sepium | nth | short | no | Hodgson |
| Capsella bursa-pastoris | awtw | medium | no | Hodgson |
| Cardamine hirsuta | nth | medium | no | Hodgson |
| Cardamine pratensis | wgdr | medium | no | Hodgson |
| Carduus crispus | nth | long | no | Wikipedia |
| Carex acuta | aqfb | short | no | authors |
| Carex hirta | wgdr | short | no | Hodgson |
| Carex otrubae | aqfb | short | yes | Hodgson |
| Carex vulpina agg. | aqfb | short | yes | Bruun |
| Centaurea cyanus | awtw | medium | no | Wikipedia |
| Centaurea jacea s.l. | m&p | medium | no | Wikipedia |
| Cerastium holosteoides | m&p | short | no | Hodgson |
| Cerastium semidecandrum | pg&h | short | no | Hodgson |
| Chaerophyllum temulum | nth | short | no | Hodgson |
| Chelidonium majus | nth | medium | no | Hodgson |
| Chenopodium album s.l. | awtw | short | no | Hodgson |
| Chenopodium polyspermum | awtw | short | yes | Floraweb |
| Cirsium arvense | nth | long | no | Hodgson |
| Cirsium palustre | wgdr | long | no | Hodgson |
| Cirsium vulgare | nth | long | no | Hodgson |
| Convolvulus arvensis | nth | short | no | Hodgson |
| Conyza canadensis | awtw | long | no | Floraweb |
| Crepis capillaris | m&p | long | no | Hodgson |
| Cynosurus cristatus | m&p | short | no | Hodgson |
| Cytisus scoparius | wood | medium | no | Hodgson |
| Dactylis glomerata | m&p | short | no | Hodgson |
| Dipsacus fullonum | nth | long | no | Floraweb |
| Dipsacus pilosus | nth | long | no | Floraweb |
| Dryopteris carthusiana | wood | long | no | Floraweb |
| Elymus repens | nth | short | no | Hodgson |
| Epilobium ciliatum | nth | long | no | Hodgson |
| Epilobium hirsutum | nth | long | no | Hodgson |
| Epilobium montanum | nth | long | no | Hodgson |
| Epilobium obscurum | aqfb | long | no | Hodgson |
| Epilobium parviflorum | aqfb | long | no | Hodgson |
| Epilobium roseum | nth | long | no | Floraweb |
| Epilobium tetragonum subsp. lamyi | nth | long | no | Floraweb |
| Epilobium tetragonum subsp. tetragonum | nth | long | no | Floraweb |
| Equisetum arvense | not assigned | long | no | Hodgson |
| Equisetum fluviatile | aqfb | long | no | Hodgson |
| Equisetum palustre | wgdr | long | no | Hodgson |
| Erodium cicutarium | pg&h | long | no | Hodgson |
| Erophila verna s.l. | pg&h | medium | no | Hodgson |
| Eupatorium cannabinum | nth | long | no | Hodgson |
| Fagopyrum esculentum | not assigned | short | no | Floraweb |
| Fagus sylvatica | wood | medium | no | Hodgson |
| Fallopia convolvulus | awtw | short | no | Hodgson |
| Fallopia dumetorum | nth | medium | no | Floraweb |
| Festuca arundinacea | wgdr | long | no | Hodgson |
| Festuca filiformis | pg&h | long | no | Hodgson |
| Festuca pratensis | m&p | long | no | Hodgson |
| Festuca rubra (s.str.) | m&p | long | no | Floraweb |
| Filipendula ulmaria | wgdr | short | yes | Hodgson |
| Frangula alnus | swood | long | no | Floraweb |
| Fraxinus excelsior | wood | medium | no | Hodgson |
| Galeopsis bifida et tetrahit | nth | short | no | Hodgson |
| Galeopsis tetrahit | nth | short | no | Hodgson |
| Galium aparine | nth | long | no | Hodgson |
| Galium mollugo agg. | m&p | long | no | Mersereau |
| Galium palustre s.l. | aqfb | short | yes | Hodgson |
| Galium palustre subsp. palustre | aqfb | short | yes | Hodgson |
| Geranium dissectum | awtw | long | no | Hodgson |
| Geranium molle | m&p | long | no | Hodgson |
| Geranium pusillum | awtw | long | no | Floraweb |
| Geranium robertianum | nth | long | no | Hodgson |
| Geum urbanum | wood | long | no | Hodgson |
| Glechoma hederacea | nth | short | no | Hodgson |
| Glyceria fluitans | aqfb | short | no | Hodgson |
| Hedera helix | wood | long | no | Hodgson |
| Helictotrichon pubescens | m&p | long | no | Hodgson |
| Heracleum sphondylium subsp. sphondylium | m&p | medium | no | Hodgson |
| Hieracium laevigatum | wood | long | no | Floraweb |
| Holcus lanatus | m&p | short | no | Hodgson |
| Holcus mollis | wood | long | no | Hodgson |
| Humulus lupulus | wood | medium | no | Hodgson |
| Hypericum maculatum s.l. | pg&h | short | no | Hodgson |
| Hypericum perforatum | nth | short | no | Hodgson |
| Hypericum tetrapterum | wgdr | short | no | Hodgson |
| Hypochaeris radicata | pg&h | long | no | Hodgson |
| Iris pseudacorus | aqfb | short | yes | Hodgson |
| Juncus acutiflorus | wgdr | long | no | Hodgson |
| Juncus articulatus | aqfb | long | no | Hodgson |
| Juncus conglomeratus | wgdr | long | no | Hodgson |
| Juncus effusus | wgdr | long | no | Hodgson |
| Juncus inflexus | wgdr | long | no | Hodgson |
| Lamium album | nth | medium | no | Hodgson |
| Lamium purpureum var. purpureum | awtw | medium | no | Hodgson |
| Lapsana communis | nth | short | no | Hodgson |
| Lathyrus pratensis | m&p | short | no | Hodgson |
| Lemna minor | aqfb | short | yes | Hodgson |
| Leontodon autumnalis subsp. autumnalis | m&p | long | no | Hodgson |
| Lolium multiflorum | awtw | long | no | Floraweb |
| Lolium perenne | m&p | long | no | Floraweb |
| Lonicera periclymenum | wood | long | no | Hodgson |
| Lotus corniculatus | m&p | short | no | Hodgson |
| Lotus pedunculatus | wgdr | short | no | Hodgson |
| Luzula campestris | pg&h | medium | no | Hodgson |
| Lycopus europaeus subsp. europaeus | aqfb | long | yes | Hodgson |
| Lysimachia nummularia | wgdr | short | no | Hodgson |
| Lysimachia vulgaris | wgdr | medium | yes | Hodgson |
| Lythrum salicaria | wgdr | long | yes | Hodgson |
| Matricaria discoidea | awtw | short | no | Hodgson |
| Matricaria recutita | awtw | long | no | Düll |
| Medicago lupulina | pg&h | short | no | Hodgson |
| Melilotus altissimus | nth | short | no | Floraweb |
| Mentha aquatica | aqfb | short | yes | Hodgson |
| Moehringia trinervia | wood | short | no | Hodgson |
| Myosotis arvensis | awtw | long | no | Hodgson |
| Myosotis scorpioides | wgdr | short | no | Hodgson |
| Nasturtium officinale agg. | aqfb | short | no | Hodgson |
| Persicaria amphibia | aqfb | short | yes | Hodgson |
| Persicaria dubia | awtw | long | yes | Floraweb |
| Persicaria hydropiper | awtw | long | yes | Floraweb |
| Persicaria lapathifolia s.l. | awtw | long | no | Floraweb |
| Persicaria maculosa | awtw | long | no | Floraweb |
| Phacelia tanacetifolia | not assigned | short | no | Floraweb |
| Phalaris arundinacea | aqfb | short | yes | Hodgson |
| Phleum pratense | m&p | long | no | Hodgson |
| Picris hieracioides s.l. | nth | long | no | Düll |
| Pimpinella major | m&p | short | no | Düll |
| Pimpinella saxifraga | pg&h | short | no | Hodgson |
| Plantago lanceolata | m&p | long | no | Hodgson |
| Plantago major s.l. | awtw | long | no | Hodgson |
| Plantago major subsp. major | awtw | long | no | Hodgson |
| Poa annua | awtw | short | no | Hodgson |
| Poa humilis | pg&h | short | no | Hodgson |
| Poa nemoralis | wood | short | no | Hodgson |
| Poa palustris | aqfb | long | no | Floraweb |
| Poa pratensis | m&p | short | no | Hodgson |
| Poa trivialis | m&p | short | no | Hodgson |
| Polygonum aviculare agg. | awtw | short | no | Hodgson |
| Populus tremula | wood | long | no | Hodgson |
| Potentilla anserina | wgdr | short | no | Hodgson |
| Potentilla erecta | pg&h | short | no | Hodgson |
| Potentilla reptans | wgdr | short | no | Hodgson |
| Primula elatior | swood | short | no | Düll |
| Prunus spinosa agg. | wood | long | no | Hodgson |
| Quercus robur | wood | medium | no | Hodgson |
| Ranunculus acris | m&p | long | no | Hodgson |
| Ranunculus ficaria subsp. bulbilifer | wood | medium | no | Hodgson |
| Ranunculus flammula | aqfb | long | yes | Hodgson |
| Ranunculus lingua | aqfb | short | yes | Düll |
| Ranunculus repens | wgdr | long | yes | Hodgson |
| Rubus caesius | swood | long | no | Hodgson |
| Rubus fruticosus agg. | wood | long | no | Hodgson |
| Rubus idaeus | wood | long | no | Hodgson |
| Rumex acetosa | m&p | medium | no | Hodgson |
| Rumex acetosella s.l. | pg&h | short | no | Hodgson |
| Rumex conglomeratus | wgdr | short | yes | Hodgson |
| Rumex crispus | wgdr | short | no | Hodgson |
| Rumex obtusifolius s.l. | nth | long | no | Hodgson |
| Rumex sanguineus | swood | short | no | Hodgson |
| Salix alba | swood | long | no | Floraweb |
| Salix cinerea s.l. | swood | long | no | Hodgson |
| Sambucus nigra | wood | long | no | Hodgson |
| Scrophularia nodosa | wood | short | no | Hodgson |
| Senecio sylvaticus | nth | long | no | Hodgson |
| Silene dioica | nth | short | no | Hodgson |
| Silene flos-cuculi | wgdr | short | no | Hodgson |
| Solanum dulcamara | aqfb | long | no | Hodgson |
| Sonchus asper | awtw | long | no | Hodgson |
| Sparganium erectum s.l. | aqfb | short | yes | Wikipedia |
| Stachys sylvatica | swood | long | no | Hodgson |
| Stellaria alsine | aqfb | short | no | Hodgson |
| Stellaria graminea | m&p | short | no | Hodgson |
| Stellaria holostea | swood | short | no | Hodgson |
| Stellaria media | awtw | short | no | Hodgson |
| Stellaria palustris | aqfb | short | no | authors |
| Symphytum officinale s.l. | nth | medium | yes | Hodgson |
| Tanacetum vulgare | nth | short | no | Hodgson |
| Taraxacum officinale agg. | m&p | long | no | Hodgson |
| Thlaspi arvense | awtw | medium | no | Hodgson |
| Tragopogon pratensis s.l. | m&p | long | no | Hodgson |
| Trifolium dubium | m&p | long | no | Hodgson |
| Trifolium hybridum s.l. | m&p | long | no | Hodgson |
| Trifolium medium | nth | long | no | Hodgson |
| Trifolium pratense | m&p | long | no | Hodgson |
| Trifolium repens | m&p | long | no | Hodgson |
| Tussilago farfara | awtw | long | no | Hodgson |
| Typha latifolia | aqfb | long | no | Hodgson |
| Urtica dioica s.l. | nth | long | no | Hodgson |
| Urtica dioica subsp. dioica | nth | long | no | Hodgson |
| Valeriana procurrens | wgdr | long | no | Hodgson |
| Veronica chamaedrys s.l. | m&p | short | no | Hodgson |
| Veronica hederifolia s.l. | awtw | medium | no | Hodgson |
| Veronica hederifolia subsp. hederifolia | awtw | medium | no | Hodgson |
| Vicia angustifolia | m&p | short | no | Hodgson |
| Vicia cracca | m&p | short | no | Hodgson |
| Vicia hirsuta | awtw | short | no | Hodgson |
| Vicia sepium | m&p | short | no | Hodgson |
| Vicia tetrasperma | awtw | short | no | Floraweb |
| Viola arvensis | awtw | short | no | Hodgson |
| Wolffia arrhiza | aqfb | long | yes | Floraweb |

**References**

Bruun HH (2009) Om flydeevnen hos frugter af Ræve-Star (*Carex vulpina*) og skovengenes forsvinden i Danmark. *Flora og Fauna* 114: 73–76.

Düll R, Kutzelnigg H (2011) *Taschenlexikon der Pflanzen Deutschlands und angrenzender Länder*. Wiebelsheim: Quelle & Meyer.

Hodgson JG, Grime JP, Hunt R, Thompson K (1995) *The electronic comparative plant ecology*. London: Chapman & Hall.

Mersereau D, DiTommaso A (2003) The biology of Canadian weeds. 121. *Galium mollugo* L. *Canadian Journal of Plant Science* 83: 453–466.
