# Supplementary material for: Effectiveness of corridors varies among phytosociological plant groups and dispersal syndromes
Source: PLoS One. 2018 Jul 11;13(7):e0199980. doi: 10.1371/journal.pone.0199980 (PMC6040708; doi:10.1371/journal.pone.0199980)
Supplement: S4 Table — Setup of Generalised Linear Mixed Models. (DOCX) [file pone.0199980.s004.docx]

Supporting information to the paper

Thiele, J., Buchholz, S. & Schirmel, J. (2018) Effectiveness of corridors varies among phytosociological plant groups and dispersal syndromes. Plos One.

**S4 Table. Model setup.** Setup of Generalised Linear Mixed Models.

**Table S3.** Descriptive statistics of the dependent variables (Jaccard similarities) and characteristics of Generalised Linear Mixed Models used for data analysis.

| **Jaccard similarity based on species group …** | **Jaccard similarity** | | | |  | **Model characteristics** | | |
| --- | --- | --- | --- | --- | --- | --- | --- | --- |
|  | **Min.** | **Mean** | **Max.** | **% zeros** |  | **Jaccard coded as** | **Over-disp.** | **Random-effect structure** |
| All species | 0.00 | 0.22 | 0.71 | 0.8 |  | proportion | yes | (1\|S)+(1\|A)+(1\|B)+(1\|pair) |
| Meadows and pastures | 0.00 | 0.29 | 0.93 | 6.1 |  | proportion | yes | (1\|S)+(1\|A)+(1\|B)+(1\|pair) |
| Aquatic communities, fens and bogs | 0.00 | 0.05 | 1.00 | 89.4 |  | model failed to converge | | |
| Arable-weed, trackside and wasteland communities | 0.00 | 0.10 | 1.00 | 77.3 |  | binary | NA | (1\|S)+(1\|A)+(1\|B) |
| Nutrient-poor grasslands and heath | 0.00 | 0.02 | 1.00 | 96.2 |  | no model for too many zeros | | |
| Tall-herb communities | 0.00 | 0.28 | 0.80 | 8.3 |  | proportion | yes | (1\|S)+(1\|A)+(1\|B)+(1\|pair) |
| Wet grasslands and dwarf rush communities | 0.00 | 0.10 | 1.00 | 68.2 |  | model failed to converge | | |
| Short-distance dispersal (non-aquatic) | 0.00 | 0.24 | 1.00 | 3.0 |  | proportion | yes | (1\|S)+(1\|A)+(1\|B)+(1\|pair) |
| Medium-distance dispersal (non-aquatic) | 0.00 | 0.18 | 1.00 | 47.7 |  | proportion | yes | (1\|S)+(1\|A)+(1\|B)+(1\|pair) |
| Long-distance dispersal (non-aquatic) | 0.00 | 0.23 | 0.83 | 2.3 |  | proportion | yes | (1\|S)+(1\|A)+(1\|B)+(1\|pair) |
| Aquatic dispersal | 0.00 | 0.14 | 1.00 | 68.9 |  | binary | NA | (1\|S)+(1\|A)+(1\|B) |
| Abbreviations in ‘random-effect structure’: S = study area; A = plot A; B = plot B; pair = individual-level random effect. Overdisp. = overdispersion | | | | | | | | |
