# Supplementary material for: Effectiveness of corridors varies among phytosociological plant groups and dispersal syndromes
Source: PLoS One. 2018 Jul 11;13(7):e0199980. doi: 10.1371/journal.pone.0199980 (PMC6040708; doi:10.1371/journal.pone.0199980)
Supplement: S5 Table — Summary tables of Generalised Linear Mixed Models. (DOCX) [file pone.0199980.s005.docx]

Supporting information to the paper

Thiele, J., Buchholz, S. & Schirmel, J. (2018) Effectiveness of corridors varies among phytosociological plant groups and dispersal syndromes. Plos One.

**S5 Table. Model summaries.** Summary tables of Generalised Linear Mixed Models.

**Tables S5a–p.** Results of Generalised Linear Mixed Models (GLMM) modelling effects of resistance distance (Tables S5a–h) and of Euclidean distance (Tables S5i–p) on Jaccard similarity of pairs of vegetation relevés from linear landscape elements (LLE), i.e. field margins and ditches, in the ‘Münsterland’ region of NW Germany. Different variants of Jaccard similarity were calculated using species of particular phytosociological groups or dispersal-distance classes, next to using all vascular plant species. The GLMM were set up with binomial distribution and logit link. Jaccard similarities were coded as proportions, except for the groups *arable-weed, trackside and wasteland communities* and *aquatic dispersal* in which it was coded as a binary variable because of many zero values (cf. Table 1). P-values from Wald z tests, Likelihood Ratio tests, and parametric bootstrap (999 iterations) are reported. Β = regression coefficient; SE_(Β)_ = standard error of regression coefficient; Z = z statistic; P(>|Z|) = p-value from Wald z test; χ² = Wald Chi squared from Type II Likelihood Ratio test; P(>χ²) = p-value from Type II Likelihood Ratio test; P_(boot)_ = p-value from parametric bootstrap. Abbreviations of predictor variables: Resistance = resistance distance (from circuit theory); LLE-types = combinations of types of LLE (field margin, ditch) in the plot pairs, with three levels: field margin with field margin, field margin with ditch, ditch with ditch.

**Table S5a.** Resistance distance – all species (cf. Fig. 2a).

| **Parameter** | **Β** | **SE_(Β)_** | **Z** | **P(>\|Z\|)** | **χ²** | **P(>χ²)** | **P_(boot)_** |
| --- | --- | --- | --- | --- | --- | --- | --- |
| Intercept | -1.365 | 0.230 | -5.930 | < 0.001 |  |  |  |
| Resistance | -1.149 | 0.229 | -5.007 | < 0.001 | 12.466 | < 0.001 | 0.001 |
| LLE-types |  |  |  |  | 4.361 | 0.113 | 0.016 |
| –margin-ditch | -0.080 | 0.163 | -0.488 | 0.626 |  |  |  |
| –ditch-ditch | 0.234 | 0.211 | 1.111 | 0.267 |  |  |  |
| Interaction:Resist.*LLE |  |  |  |  | 16.751 | < 0.001 | 0.001 |
| –Resist.*margin-ditch | 0.942 | 0.247 | 3.818 | < 0.001 |  |  |  |
| –Resist.*ditch-ditch | 1.097 | 0.276 | 3.983 | < 0.001 |  |  |  |
| Random effects (standard deviations): study area 0.353, plot A 0.000, plot B 0.000, pair 0.487 | | | | | | | |

**Table S5b.** Resistance distance – meadow and pasture species (cf. Fig. 2b).

| **Parameter** | **Β** | **SE(Β)** | **Z** | **P(>\|Z\|)** | **χ²** | **P(>χ²)** | **P(boot)** |
| --- | --- | --- | --- | --- | --- | --- | --- |
| Intercept | -1.191 | 0.382 | -3.116 | 0.002 |  |  |  |
| Resistance | -1.701 | 0.447 | -3.803 | < 0.001 | 7.962 | 0.005 | 0.002 |
| LLE-types |  |  |  |  | 0.435 | 0.804 | 0.489 |
| –margin-ditch | 0.076 | 0.320 | 0.238 | 0.812 |  |  |  |
| –ditch-ditch | 0.269 | 0.415 | 0.647 | 0.518 |  |  |  |
| Interaction:Resist.*LLE |  |  |  |  | 10.465 | 0.005 | 0.001 |
| –Resist.*margin-ditch | 1.310 | 0.478 | 2.742 | 0.006 |  |  |  |
| –Resist.*ditch-ditch | 1.723 | 0.533 | 3.230 | 0.001 |  |  |  |
| Random effects (standard deviations): study area 0.497, plot A 0.000, plot B 0.000, pair 1.007 | | | | | | | |

**Table S5c.** Resistance distance – species of tall-herb communities (cf. Fig. 2c).

| **Parameter** | **Β** | **SE_(Β)_** | **Z** | **P(>\|Z\|)** | **χ²** | **P(>χ²)** | **P_(boot)_** |
| --- | --- | --- | --- | --- | --- | --- | --- |
| Intercept | -1.321 | 0.466 | -2.833 | 0.005 |  |  |  |
| Resistance | -0.655 | 0.456 | -1.436 | 0.151 | 1.935 | 0.164 | 0.062 |
| LLE-types |  |  |  |  | 6.501 | 0.039 | 0.002 |
| –margin-ditch | -0.107 | 0.330 | -0.324 | 0.746 |  |  |  |
| –ditch-ditch | 0.813 | 0.426 | 1.909 | 0.056 |  |  |  |
| Interaction:Resist.*LLE |  |  |  |  | 1.065 | 0.587 | 0.233 |
| –Resist.*margin-ditch | 0.463 | 0.491 | 0.944 | 0.345 |  |  |  |
| –Resist.*ditch-ditch | 0.558 | 0.551 | 1.013 | 0.311 |  |  |  |
| Random effects (standard deviations): study area 0.713, plot A 0.000, plot B 0.000, pair 1.036 | | | | | | | |

**Table S5d.** Resistance distance – species of arable-weed, trackside and wasteland communities (cf. Fig. 2d).

| **Parameter** | **Β** | **SE_(Β)_** | **Z** | **P(>\|Z\|)** | **χ²** | **P(>χ²)** | **P_(boot)_** |
| --- | --- | --- | --- | --- | --- | --- | --- |
| Intercept | -2.679 | 1.192 | -2.248 | 0.025 |  |  |  |
| Resistance | -1.440 | 1.300 | -1.108 | 0.268 | 2.069 | 0.150 | 0.021 |
| LLE-types |  |  |  |  | 0.127 | 0.939 | 0.927 |
| –margin-ditch | 0.462 | 0.919 | 0.503 | 0.615 |  |  |  |
| –ditch-ditch | 0.286 | 1.420 | 0.201 | 0.840 |  |  |  |
| Interaction:Resist.*LLE |  |  |  |  | 1.075 | 0.584 | 0.305 |
| –Resist.*margin-ditch | 0.960 | 1.372 | 0.700 | 0.484 |  |  |  |
| –Resist.*ditch-ditch | -0.167 | 1.782 | -0.094 | 0.925 |  |  |  |
| Random effects (standard deviations): study area 0.653, plot A 1.481, plot B 1.481 | | | | | | | |

**Table S5e.** Resistance distance – species with short-distance dispersal (non-aquatic dispersal modes; cf. Fig. 3a).

| **Parameter** | **Β** | **SE_(Β)_** | **Z** | **P(>\|Z\|)** | **χ²** | **P(>χ²)** | **P_(boot)_** |
| --- | --- | --- | --- | --- | --- | --- | --- |
| Intercept | -1.357 | 0.295 | -4.601 | < 0.001 |  |  |  |
| Resistance | -1.585 | 0.361 | -4.397 | < 0.001 | 12.614 | < 0.001 | 0.001 |
| LLE-types |  |  |  |  | 2.063 | 0.357 | 0.106 |
| –margin-ditch | -0.028 | 0.256 | -0.108 | 0.914 |  |  |  |
| –ditch-ditch | 0.342 | 0.332 | 1.032 | 0.302 |  |  |  |
| Interaction:Resist.*LLE |  |  |  |  | 11.907 | 0.003 | 0.001 |
| –Resist.*margin-ditch | 1.209 | 0.384 | 3.151 | 0.002 |  |  |  |
| –Resist.*ditch-ditch | 1.456 | 0.428 | 3.404 | 0.001 |  |  |  |
| Random effects (standard deviations): study area 0.363, plot A 0.000, plot B 0.000, pair 0.801 | | | | | | | |

**Table S5f.** Resistance distance – species with medium-distance dispersal (non-aquatic dispersal modes; cf. Fig. 3b).

| **Parameter** | **Β** | **SE_(Β)_** | **Z** | **P(>\|Z\|)** | **χ²** | **P(>χ²)** | **P_(boot)_** |
| --- | --- | --- | --- | --- | --- | --- | --- |
| Intercept | -4.902 | 1.671 | -2.933 | 0.003 |  |  |  |
| Resistance | -5.614 | 2.034 | -2.760 | 0.006 | 1.498 | 0.221 | 0.165 |
| LLE-types |  |  |  |  | 3.126 | 0.210 | 0.027 |
| –margin-ditch | -1.112 | 1.445 | -0.769 | 0.442 |  |  |  |
| –ditch-ditch | 1.798 | 1.860 | 0.966 | 0.334 |  |  |  |
| Interaction:Resist.*LLE |  |  |  |  | 7.539 | 0.023 | 0.001 |
| –Resist.*margin-ditch | 5.763 | 2.179 | 2.645 | 0.008 |  |  |  |
| –Resist.*ditch-ditch | 4.022 | 2.427 | 1.657 | 0.098 |  |  |  |
| Random effects (standard deviations): study area 2.033, plot A 0.000, plot B 0.000, pair 4.070 | | | | | | | |

**Table S5g.** Resistance distance – species with long-distance dispersal (non-aquatic dispersal modes; cf. Fig. 3c).

| **Parameter** | **Β** | **SE_(Β)_** | **Z** | **P(>\|Z\|)** | **χ²** | **P(>χ²)** | **P_(boot)_** |
| --- | --- | --- | --- | --- | --- | --- | --- |
| Intercept | -1.330 | 0.281 | -4.733 | < 0.001 |  |  |  |
| Resistance | -0.684 | 0.330 | -2.074 | 0.038 | 1.012 | 0.314 | 0.200 |
| LLE-types |  |  |  |  | 2.217 | 0.330 | 0.093 |
| –margin-ditch | -0.124 | 0.236 | -0.525 | 0.600 |  |  |  |
| –ditch-ditch | 0.224 | 0.306 | 0.730 | 0.465 |  |  |  |
| Interaction:Resist.*LLE |  |  |  |  | 3.964 | 0.138 | 0.016 |
| –Resist.*margin-ditch | 0.595 | 0.354 | 1.683 | 0.092 |  |  |  |
| –Resist.*ditch-ditch | 0.786 | 0.395 | 1.989 | 0.047 |  |  |  |
| Random effects (standard deviations): study area 0.365, plot A 0.000, plot B 0.000, pair 0.738 | | | | | | | |

**Table S5h.** Resistance distance – species with aquatic dispersal modes (cf. Fig. 3d).

| **Parameter** | **Β** | **SE_(Β)_** | **Z** | **P(>\|Z\|)** | **χ²** | **P(>χ²)** | **P_(boot)_** |
| --- | --- | --- | --- | --- | --- | --- | --- |
| Intercept | -0.832 | 0.858 | -0.970 | 0.332 |  |  |  |
| Resistance | -1.674 | 0.960 | -1.743 | 0.081 | 1.563 | 0.211 | 0.063 |
| LLE-types |  |  |  |  | 3.171 | 0.205 | 0.004 |
| –margin-ditch | -0.732 | 0.762 | -0.961 | 0.337 |  |  |  |
| –ditch-ditch | 0.583 | 0.893 | 0.653 | 0.514 |  |  |  |
| Interaction:Resist.*LLE |  |  |  |  | 2.174 | 0.337 | 0.130 |
| –Resist.*margin-ditch | 1.451 | 1.003 | 1.447 | 0.148 |  |  |  |
| –Resist.*ditch-ditch | 1.178 | 1.100 | 1.072 | 0.284 |  |  |  |
| Random effects (standard deviations): study area 1.130, plot A 0.849, plot B 0.849 | | | | | | | |

**Table S5i.** Euclidean distance – all species.

| **Parameter** | **Β** | **SE_(Β)_** | **Z** | **P(>\|Z\|)** | **χ²** | **P(>χ²)** | **P_(boot)_** |
| --- | --- | --- | --- | --- | --- | --- | --- |
| Intercept | -1.273 | 0.205 | -6.205 | < 0.001 |  |  |  |
| Euclidean distance | -0.439 | 0.190 | -2.313 | 0.021 | 3.298 | 0.069 | 0.016 |
| LLE-types |  |  |  |  | 3.894 | 0.143 | 0.028 |
| –margin-ditch | -0.206 | 0.189 | -1.091 | 0.275 |  |  |  |
| –ditch-ditch | 0.139 | 0.246 | 0.567 | 0.571 |  |  |  |
| Interaction:Eucl. dist.*LLE |  |  |  |  | 3.420 | 0.181 | 0.027 |
| –Eucl. dist.*margin-ditch | 0.329 | 0.218 | 1.508 | 0.132 |  |  |  |
| –Eucl. dist.*ditch-ditch | 0.461 | 0.257 | 1.796 | 0.073 |  |  |  |
| Random effects (standard deviations): study area 0.231, plot A 0.000, plot B 0.000, pair 0.589 | | | | | | | |

**Table S5j.** Euclidean distance – meadow and pasture species.

| **Parameter** | **Β** | **SE(Β)** | **Z** | **P(>\|Z\|)** | **χ²** | **P(>χ²)** | **P(boot)** |
| --- | --- | --- | --- | --- | --- | --- | --- |
| Intercept | -1.079 | 0.351 | -3.074 | 0.002 |  |  |  |
| Euclidean distance | -0.632 | 0.364 | -1.738 | 0.082 | 2.120 | 0.145 | 0.048 |
| LLE-types |  |  |  |  | 0.574 | 0.750 | 0.501 |
| –margin-ditch | -0.110 | 0.360 | -0.304 | 0.761 |  |  |  |
| –ditch-ditch | 0.154 | 0.468 | 0.329 | 0.742 |  |  |  |
| Interaction:Eucl. dist.*LLE |  |  |  |  | 1.693 | 0.429 | 0.130 |
| –Eucl. dist.*margin-ditch | 0.467 | 0.413 | 1.129 | 0.259 |  |  |  |
| –Eucl. dist.*ditch-ditch | 0.598 | 0.487 | 1.228 | 0.220 |  |  |  |
| Random effects (standard deviations): study area 0.285, plot A 0.000, plot B 0.000, pair 1.161 | | | | | | | |

**Table S5k.** Euclidean distance – species of tall-herb communities.

| **Parameter** | **Β** | **SE_(Β)_** | **Z** | **P(>\|Z\|)** | **χ²** | **P(>χ²)** | **P_(boot)_** |
| --- | --- | --- | --- | --- | --- | --- | --- |
| Intercept | -1.327 | 0.440 | -3.013 | 0.003 |  |  |  |
| Euclidean distance | -0.501 | 0.328 | -1.528 | 0.127 | 2.082 | 0.149 | 0.046 |
| LLE-types |  |  |  |  | 7.035 | 0.030 | 0.003 |
| –margin-ditch | -0.122 | 0.327 | -0.374 | 0.709 |  |  |  |
| –ditch-ditch | 0.819 | 0.425 | 1.926 | 0.054 |  |  |  |
| Interaction:Eucl. dist.*LLE |  |  |  |  | 1.333 | 0.514 | 0.196 |
| –Eucl. dist.*margin-ditch | 0.327 | 0.378 | 0.865 | 0.387 |  |  |  |
| –Eucl. dist.*ditch-ditch | 0.508 | 0.444 | 1.144 | 0.253 |  |  |  |
| Random effects (standard deviations): study area 0.652, plot A 0.000, plot B 0.000, pair 1.043 | | | | | | | |

**Table S5l.** Euclidean distance – species of arable-weed, trackside and wasteland communities.

| **Parameter** | **Β** | **SE_(Β)_** | **Z** | **P(>\|Z\|)** | **χ²** | **P(>χ²)** | **P_(boot)_** |
| --- | --- | --- | --- | --- | --- | --- | --- |
| Intercept | -2.272 | 1.051 | -2.162 | 0.031 |  |  |  |
| Euclidean distance | 0.385 | 0.789 | 0.488 | 0.626 | 0.688 | 0.407 | 0.231 |
| LLE-types |  |  |  |  | 0.080 | 0.961 | 0.929 |
| –margin-ditch | -0.010 | 0.862 | -0.012 | 0.991 |  |  |  |
| –ditch-ditch | 0.233 | 1.294 | 0.180 | 0.857 |  |  |  |
| Interaction:Eucl. dist.*LLE |  |  |  |  | 0.906 | 0.636 | 0.414 |
| –Eucl. dist.*margin-ditch | -0.852 | 0.896 | -0.951 | 0.342 |  |  |  |
| –Eucl. dist.*ditch-ditch | -0.677 | 1.089 | -0.622 | 0.534 |  |  |  |
| Random effects (standard deviations): study area 0.442, plot A 1.589, plot B 1.589 | | | | | | | |

**Table S5m.** Euclidean distance – species with short-distance dispersal (non-aquatic dispersal modes).

| **Parameter** | **Β** | **SE_(Β)_** | **Z** | **P(>\|Z\|)** | **χ²** | **P(>χ²)** | **P_(boot)_** |
| --- | --- | --- | --- | --- | --- | --- | --- |
| Intercept | -1.291 | 0.258 | -5.003 | 0.000 |  |  |  |
| Euclidean distance | -0.777 | 0.289 | -2.692 | 0.007 | 4.208 | 0.040 | 0.010 |
| LLE-types |  |  |  |  | 2.284 | 0.319 | 0.107 |
| –margin-ditch | -0.160 | 0.287 | -0.559 | 0.576 |  |  |  |
| –ditch-ditch | 0.270 | 0.369 | 0.730 | 0.465 |  |  |  |
| Interaction:Eucl. dist.*LLE |  |  |  |  | 5.164 | 0.076 | 0.005 |
| –Eucl. dist.*margin-ditch | 0.562 | 0.326 | 1.723 | 0.085 |  |  |  |
| –Eucl. dist.*ditch-ditch | 0.871 | 0.387 | 2.248 | 0.025 |  |  |  |
| Random effects (standard deviations): study area 0.134, plot A 0.000, plot B 0.000, pair 0.926 | | | | | | | |

**Table S5n.** Euclidean distance – species with medium-distance dispersal (non-aquatic dispersal modes).

| **Parameter** | **Β** | **SE_(Β)_** | **Z** | **P(>\|Z\|)** | **χ²** | **P(>χ²)** | **P_(boot)_** |
| --- | --- | --- | --- | --- | --- | --- | --- |
| Intercept | -4.023 | 1.617 | -2.489 | 0.013 |  |  |  |
| Euclidean distance | -0.850 | 1.623 | -0.524 | 0.601 | 1.131 | 0.288 | 0.176 |
| LLE-types |  |  |  |  | 3.951 | 0.139 | 0.027 |
| –margin-ditch | -2.125 | 1.592 | -1.335 | 0.182 |  |  |  |
| –ditch-ditch | 0.939 | 1.985 | 0.473 | 0.636 |  |  |  |
| Interaction:Eucl. dist.*LLE |  |  |  |  | 0.486 | 0.784 | 0.655 |
| –Eucl. dist.*margin-ditch | 0.432 | 1.865 | 0.232 | 0.817 |  |  |  |
| –Eucl. dist.*ditch-ditch | -0.798 | 2.196 | -0.363 | 0.716 |  |  |  |
| Random effects (standard deviations): study area 1.642, plot A 0.000, plot B 0.000, pair 4.351 | | | | | | | |

**Table S5o.** Euclidean distance – species with long-distance dispersal (non-aquatic dispersal modes).

| **Parameter** | **Β** | **SE_(Β)_** | **Z** | **P(>\|Z\|)** | **χ²** | **P(>χ²)** | **P_(boot)_** |
| --- | --- | --- | --- | --- | --- | --- | --- |
| Intercept | -1.288 | 0.263 | -4.890 | 0.000 |  |  |  |
| Euclidean distance | -0.398 | 0.240 | -1.656 | 0.098 | 0.446 | 0.504 | 0.365 |
| LLE-types |  |  |  |  | 2.332 | 0.312 | 0.091 |
| –margin-ditch | -0.179 | 0.239 | -0.749 | 0.454 |  |  |  |
| –ditch-ditch | 0.179 | 0.312 | 0.575 | 0.565 |  |  |  |
| Interaction:Eucl. dist.*LLE |  |  |  |  | 2.425 | 0.298 | 0.072 |
| –Eucl. dist.*margin-ditch | 0.387 | 0.276 | 1.401 | 0.161 |  |  |  |
| –Eucl. dist.*ditch-ditch | 0.467 | 0.327 | 1.431 | 0.153 |  |  |  |
| Random effects (standard deviations): study area 0.306, plot A 0.000, plot B 0.000, pair 0.760 | | | | | | | |

**Table S5p.** Euclidean distance – species with aquatic dispersal modes.

| **Parameter** | **Β** | **SE_(Β)_** | **Z** | **P(>\|Z\|)** | **χ²** | **P(>χ²)** | **P_(boot)_** |
| --- | --- | --- | --- | --- | --- | --- | --- |
| Intercept | -0.638 | 0.859 | -0.742 | 0.458 |  |  |  |
| Euclidean distance | -0.312 | 0.674 | -0.463 | 0.643 | 0.783 | 0.376 | 0.201 |
| LLE-types |  |  |  |  | 3.835 | 0.147 | 0.002 |
| –margin-ditch | -1.116 | 0.819 | -1.363 | 0.173 |  |  |  |
| –ditch-ditch | 0.395 | 1.004 | 0.394 | 0.694 |  |  |  |
| Interaction:Eucl. dist.*LLE |  |  |  |  | 0.714 | 0.700 | 0.494 |
| –Eucl. dist.*margin-ditch | 0.215 | 0.769 | 0.279 | 0.780 |  |  |  |
| –Eucl. dist.*ditch-ditch | -0.396 | 0.920 | -0.431 | 0.667 |  |  |  |
| Random effects (standard deviations): study area 0.970, plot A 1.113, plot B 1.113 | | | | | | | |
